# Comparative oncology using domesticated dogs and their microbiome

**DOI:** 10.3389/fvets.2024.1378551

**Published:** 2024-03-28

**Authors:** Tasha M. Santiago-Rodriguez

**Affiliations:** Diversigen Inc., New Brighton, MN, United States

**Keywords:** cancer, cancer treatment, canine models, comparative oncology, gut microbiome

## Introduction

A significant part of translational research on human diseases relies on the use of animal models, and mouse or murine models of specific diseases are some of the most commonly used (1). Murine models have several advantages over other animal models in translational research including short gestation times, relatively inexpensive and easy to maintain compared to other animal models. In addition, technologies have been developed to manipulate specific gene expression patterns in selected cells and/or tissues that enable the development of specific diseases in murine models. Using mouse models in translational research, however, is not without limitations. For instance, while humans develop specific disease states in a spontaneous manner, diseases in mouse models need to be induced or developed genetically (2). In addition, while genetic manipulations in mice often seem to involve one or a few genes, as well as specific environmental conditions that affect gene expression, some of these genes may or may not be relevant to specific human diseases (1).

For the abovementioned reasons, alternative animal models are increasingly being considered to study and understand human diseases. Particularly, domesticated or pet dogs (i.e., *Canis lupus familiaris*) are increasingly being recognized as models of complex human diseases for several reasons (1). For instance, next to humans, domesticated dogs have the most phenotypic diversity, and approximately 400 inherited diseases are similar between humans and dogs (3). Some of these disease states include heart disease, neurological disorders, and cancers, and more than 40 naturally occurring canine diseases have genetic mutations in a homologous human gene associated with a similar disease phenotype (4). In addition, it is estimated that over 70 million dogs live in around 40% of USA households, and 54% of those dogs are considered a family member; thus, dog health care is second to human health care in the level of care received.

## Comparative oncology: from murine to canine translational models

Cancer is among the most complex diseases affecting humans as it may be multifactorial in nature (1). Understanding cancer and developing cancer therapeutics come in hand with the use of translational animal models. Also known as comparative oncology, it is an area that is quickly expanding to examine and compare the development of cancers across animal species (5). As mentioned above, murine models are used in translational research, including cancer; yet, mouse models of cancer in humans may not cover the vast and possibly complex gene network and interactions that may be responsible for or contribute to cancers in humans (6). Due to the genetic similarities between dogs and humans, pet dogs are, and are increasingly being considered, more suitable research models compared to mice for several diseases, including various types of cancers. While specific diseases need to be induced in mouse models, dogs can naturally develop diseases, including cancers, in a manner very similar to humans. For instance, dog tumors can be histologically similar to those in humans and may respond similarly to cancer therapy (6). This similarity to develop diseases between humans and dogs has mostly been associated with a biological and genetic component, where specific mutations, deletions and insertions have been observed in both humans and dogs, resulting in similar phenotypic changes (6). These genetic similarities between humans and dogs associated with various cancers also aid in the development of therapeutics that may be used both in dogs and humans.

Cancer drugs, however, pose one of the greatest challenge to enter human clinical trials, with approximately 3.4%−6.4% of those drugs advancing successfully (7). Translational animal models can be used to decrease these failure rates of cancers drugs entering human clinical trials. In addition, translational animal models may be used to assess and potentially reduce costs and time associated with developing and testing new cancer therapeutics. Importantly, the efficacy and safety of cancer drugs may be validated in translational animal models that reflect the complexities of the human disease, including comorbidities and other factors (e.g., diet). Most preclinical immunotherapy studies using mouse models have translated poorly due to intrinsic genetic and immunological differences between mice and human (7). For this reason, animal models, such as pet dogs have been used to develop cancer treatment drugs, which have successfully entered clinical trials. It should be noted, however, that differences in pharmacodynamics have been observed across dog breeds (8). Since many breed-specific features are known to affect drug absorption and metabolism, cancer studies and clinical trials should be limited to a specific dog breed, when possible. In addition, all domestic dog breeds lack specific genes that detoxify many drugs and metabolites, which may be present in humans (8). Although canine pharmacodynamics and metabolism are not a perfect translational model, valid conclusions can still be drawn from canines as models of cancer and therapeutics in humans. In addition, drug metabolism and treatment efficiency may be affected by other factors, particularly the gut microbiome (6), part of which is also known to be shared across humans and dogs (9).

## Similarities between human and canine microbiomes in health and cancer

Bacteria present in the canine gut are known to be more similar to and shared in higher proportions with those present in the human gut compared to mouse and pig gut microbiomes (9). At the phylum level, for instance, the human and dog gut microbiomes cluster more closely compared to the mouse and pig gut microbiomes, and a median of approximately 60%−65% of the bacterial sequences are shared between human and canine gut microbiomes (9). This degree of similarity between human and dog gut microbiomes may be in part due to household sharing. While most of the composition of the human and animal gut microbiota seemed to be influenced by diet and disease states (9), household is also known to influence the level of shared microbes. For instance, dog housemates have shown similar gut and skin microbiome profiles to those of the human(s) sharing the same space compared to other dogs (10, 11).

Humans and dogs do not only share their microbiota, but gut microbial changes associated with disease states, including colorectal cancer (discussed below), have shown to be comparable between both species. Colorectal tumors occur spontaneously in dogs, and disease progression and pathogenesis seem to mimic those in humans in a genetic and microbial level. At a microbial level, particularly, the intestinal microbial community structure in dogs with tumors is different from that of healthy, control groups (12). When using high-throughput sequencing of the 16S *rRNA* gene (16S), the group of dogs with tumors is characterized by a higher representation of *Enterobacteriaceae, Bacteroides, Helicobacter, Porphyromonas, Peptostreptococcus*, and *Streptococcus*, and a lower abundance of *Ruminococcaceae, Slackia, Clostridium* XI, and *Faecalibacterium* (12). Interestingly, some of these bacteria have also been identified as potential contributors to human colorectal tumorigenesis (13–15). In addition to the gut microbiota, the oral microbiota seems to also have a role in colorectal cancer progression. For instance, an overrepresentation of oral bacteria, including *Fusobacterium, Peptostreptococcus*, and *Porphyromonas* in the fecal microbiota has been observed in both humans and dogs with colorectal tumors (16). These preliminary studies aid in the planning of future studies with potentially breed- and age-matched case-controls to evaluate the impact of the intestinal microbiota on canine colorectal progression.

Other studies have been performed to understand the associations between the dog microbiome and other types of cancers. One study looked at the gut microbiomes of dogs diagnosed with advanced stages of a specific type of lymphoma, and those of healthy dogs (17). Lymphoma affects 20–100 per 100,000 dogs annually and may have a survival span of approximately a year (18). Since the gastrointestinal tract is a common site for specific types of lymphoma, insights into the gut microbiota may reveal its potential role(s) in the disease progression and/or how this cancer type may potentially affect the gut microbiota structure and function. It is known that the microbiota may impact the efficiency of cancer treatments; thus, characterizing microbial communities in association with lymphoma and other types of cancers can also help predict the outcome of therapy (i.e., responders vs. non-responders) (19). This particular study analyzed the gut microbiome of dogs from veterinary hospitals diagnosed with lymphoma and healthy counterparts (17). By using 16S sequencing of the V3–V4 regions, several bacterial taxa were identified to be over- or under-represented in the disease vs. healthy groups. While several species such as *Corynebacterium amycolatum, Peptostreptococcus canis*, and *Proteus mirabilis* were significantly more represented in dogs with lymphoma, and *Blautia schinkii*, [*Clostridium*] *spiroforme*, and *Roseburia intestinalis* were significantly more represented in healthy dogs (17), 16S sequencing of specific regions can provide limited taxonomic classification, usually up to the genus level, and no functional information is provided, unless imputed.

Another study looked at the gut and oral microbiomes of healthy dogs and those with canine mammary tumors (CMT) (20). As with other cancer types, mammary gland tumors are similar between humans and dogs; thus, CMT in dogs may potentially serve as a translational model for human breast cancer studies. Tumors are multifactorial in nature, but certain tumors are known to be home to specific intracellular bacteria. Breast, lung, ovary, pancreas, melanoma, bone, and brain tumors seem to possess their own distinct microbiome profiles, and breast cancer, particularly, has a rich and diverse microbiome (21). Some of these bacteria in tumors are hypothesized to migrate from the gut through the bloodstream and cause disease (22). Interestingly, oral dysbiosis has also been associated with the development of certain tumors (23). These studies support that oral and gut microbiome dysbioses are associated with tumorigenesis. By using 16S sequencing, the study by Zheng et al. (20) showed that the gut microbial profiles had specific taxa over- and under-represented in health or in association with CMT. For instance, *Bacteroides* seems to be over-represented, while *Ralstonia* seems to be under-represented in the gut microbiome of dogs with CMT. Tumors also seemed to possess a lower alpha diversity compared to tissue from healthy dogs, and a unique microbiota (20).

## The needle in the haystack of comparative oncology: why more comprehensive microbiome studies using dogs are needed?

Canine cancer studies and the potential associations with the gut microbiome are still scarce and limited to 16S amplicon sequencing. Unlike shotgun metagenomics, 16S amplicon sequencing does not provide species and strains level resolution needed to potentially understand cancer progression, develop treatments, and predict treatment response. In addition, metabolic pathways potentially associated with the progression of various cancers can only be elucidated through shotgun metagenomics. Thus, more untargeted sequencing approaches are needed to understand the canine gut microbiome in relation to health and cancer, and how it may potentially be compared to that of humans under similar conditions.

Identification of key species, strains and metabolic pathways may depend, in part, by host-specific databases. Using reference databases composed of mainly human-relevant microbes in canine studies are known to provide limited information on bacterial taxa since species level resolution usually cannot be achieved, and a large proportion of the gut microbiome bacterial components and their potential functions cannot be assigned. Canine databases can be developed using gut-specific microbial genomes and microbial assembled genomes (MAGs), which in theory, should improve the identification of key microbial taxa and their associated functions. Thus, access to representative canine gut samples of various cancer states may represent an opportunity to develop canine-specific microbiome databases that should enable the identification of important taxa and associated potential metabolic functions and how this information may potentially translate to human oncology and cancer therapy.

## Limitations of comparative oncology studies using canine gut microbiomes

While evidence suggests that humans and dogs share similar genetic and pathway modifications that may lead to cancer (24), and approximately 60%−65% of gut bacteria (9), comparative oncology using canine gut microbiomes is a vastly unexplored area, and thus it is not expected to be without limitations that may result in lack of consistency across studies. Several of these drawbacks may be due in part to the intrinsic complexities associated with cancer, and differences in microbiome composition between humans and dogs. For instance, studies exploring dysbiosis in dogs have not conclusively pinpoint gut microbiome signature changes associated with specific diseases or drugs, even though the Dysbiosis Index became a validated quantitative PCR assay (25). Similarly, studies exploring the canine oral microbiome have not identified key taxa and their role in disease progression, and different phyla have been associated with periodontal disease in dogs and humans (11).

Differences in study design, and data generation and analysis are also factors that may influence the lack of consistency across comparative gut microbiome studies. For instance, while sample collection and storage, nucleic acid extraction, sequencing approach (amplicon vs. shotgun metagenomics), variable region (as in the case of 16S sequencing), and data processing and analysis are factors that influence human gut microbiome studies (26), it is unknown how these factors influence canine gut microbiome studies. Therefore, consistency across and between canine and human gut microbiome studies from sample collection to data analysis will be essential when performing comparative oncology studies to determine if inconsistencies are due to microbiome differences, or biases associated with the mentioned factors.

## Author contributions

TS-R: Writing – original draft, Writing – review & editing.
